# Benedikt on Brains of Criminals

**Published:** 1881-12

**Authors:** 


*Benedikt on Brains of Criminals.* Translated from the German by E. P. Fowler, M. D. Publishers: William Wood & Co. New York City, 1881.

This work consists of anatomical studies upon the brains of criminals, being a contribution to Atnhropology, Medicine, Jurisprudence, and Psychology, by Moritz Benedikt, Professor at Vienna. In view of the exciting trial now progressing in Washington City, this work is an interesting one, and affords some knowledge of a subject concerning which there has long existed a lack of material. The author asserts that crime is in no way analogous to monomania, and that it results from the physical organization as a unit, and its particular form of expression is determined by social circumstances.

